# Effects of a Structured Resistance Training Program on Muscular Strength and Functional Performance in Children with Autism Spectrum Disorder: A 12-Week Intervention Study

**DOI:** 10.3390/children13070845

**Published:** 2026-06-23

**Authors:** Janhavi Nowbotsing, Petro Erasmus, Mariaan van Aswegen

**Affiliations:** 1 Physical Activity, Sport and Recreation (PhASRec) Research Focus Area, North-West University, Potchefstroom 2531, South Africa; janvinowbotsing@gmail.com; 2 Community Psychosocial Research (COMPRES), Psychology, North-West University, Mafikeng Campus, Mmabatho 2735, South Africa; petro.erasmus@nwu.ac.za

**Keywords:** autism spectrum disorder, resistance training, children, muscle strength, functional mobility, physical activity

## Abstract

**Highlights:**

**What are the main findings?**

• A 12-week resistance training program improved lower-body power in children with mild Autism spectrum disorder.
• Functional walking performance improved over time following participation in structured exercise training.

**What are the implications of the main findings?**

• Resistance training is a feasible and safe intervention for improving physical function in children with Autism spectrum disorder.
• Structured exercise programs may support participation and physical development in children with developmental conditions.

**Abstract:**

**Background/Objectives:** Motor impairments, including reduced muscular strength and coordination, are commonly reported in children with autism spectrum disorder (ASD) and may negatively affect functional mobility and participation in daily activities. Despite increasing recognition of these challenges, structured resistance training programs for children with ASD remain limited. This study aimed to examine the effects of a 12-week resistance training program on muscular strength and functional performance in children aged 9–11 years with mild ASD. **Methods:** A selected-group repeated-measures design was employed. Twenty-eight children with specialist-confirmed mild ASD were allocated to an exercise (*n* = 14) or control group (*n* = 14) using a strength-matched allocation procedure. The intervention followed established exercise guidelines for youth. Assessments were conducted at baseline, week 6, and week 12 and included handgrip strength, vertical jump height, and 10-m walk time. Non-parametric Friedman tests assessed changes over time, followed by Durbin–Conover post hoc comparisons where appropriate. Effect sizes (r) were calculated. **Results:** No significant overall time effect was observed for handgrip strength, although a between-group difference favoring the exercise group was observed at week 6. Vertical jump height demonstrated a significant effect over time, with improvements observed in the exercise group from baseline to week 6 and a between-group difference at week 6. Walking time improved significantly across the study period, with improvements observed in both the exercise and control groups. **Conclusions:** These findings suggest that structured resistance training is a feasible intervention that may support improvements in physical function in children with mild ASD. Resistance training may therefore represent a useful component of exercise programs aimed at improving functional mobility and participation in children with developmental conditions.

## 1. Introduction

Autism spectrum disorder (ASD) is a heterogeneous neurodevelopmental condition characterized by impairments in social interaction, repetitive behaviors, and motor dysfunction, including reduced coordination, hypotonia, and balance deficits [1,2,3]. This disorder involves the abnormal functioning of the central nervous system (CNS), thus affecting multiple aspects of daily functioning and quality of life (QoL), including gait, sensory processing, and motor control [4,5,6]. ASD presents along a spectrum of severity, with functional impact varying according to symptom severity and adaptive capacity [2,6,7,8]. Symptom severity may remain stable or worsen with time [9]. Early identification of ASD is considered important for facilitating access to appropriate interventions and support structures for both the individual and their caregivers [9,10,11,12,13].

Exercise-based interventions are increasingly recognized as non-pharmacological strategies to support physical, cognitive, and behavioral functioning in children with ASD [3,14]. Physical activity broadly refers to any bodily movement that increases energy expenditure, whereas exercise is defined as planned, structured, and repetitive activity performed with the objective of improving specific components of physical fitness [3,14]. Within exercise-based interventions, important conceptual distinctions exist between aerobic training and resistance training. Aerobic exercise primarily targets cardiorespiratory fitness and has been associated with improvements in attention, behavioral regulation, and endurance in children with ASD [14,15,16]. In contrast, resistance training focuses on improving muscular strength, power, and neuromuscular control, domains frequently compromised in children with ASD due to delayed motor development, hypotonia, and reduced postural stability [3,17,18]. Structured exercise programs are increasingly recognized as important non-pharmacological strategies to improve physical fitness and functional performance in children with ASD [1,2,15,17]. Participation in physical activity has been associated with improvements in motor skill performance, muscular strength, sensory integration, behavioral regulation, attention, communication skills, and academic performance [4,18,19,20]. Despite substantial evidence supporting aerobic and general physical activity interventions in children with ASD, resistance training remains comparatively under-investigated. This represents a critical gap, as resistance training specifically targets muscular strength, power, and neuromuscular control domains that are frequently impaired in this population. Consequently, there is a need for structured, well-defined resistance training interventions to better understand their potential role in improving functional performance in pediatric ASD populations [21,22,23]. From a developmental perspective, the preadolescent period (9–11 years) represents a critical window for neuromuscular adaptation, during which strength gains are predominantly driven by neural mechanisms rather than muscle hypertrophy. This makes resistance training particularly relevant for children with ASD, who commonly present with neuromuscular inefficiencies. The present study therefore focused on children aged 9–11 years to examine responsiveness to a structured resistance training intervention during this sensitive developmental phase [18,24,25,26].

Resistance training involves the activation of skeletal muscles against an external force, such as body weight, free weights, machines, or elastic bands [3,18,27]. This, in turn, has many health benefits, including increasing bone mass density and the basal metabolic rate, promoting glucose metabolism and weight management, and reducing body mass index [22,23]. Despite these potential benefits, there remains a lack of studies systematically examining structured resistance training interventions in children with ASD. Consequently, further investigation is required to better understand the role of resistance training in supporting physical function in this population.

Children with mild ASD were selected to improve task comprehension and program adherence, thereby reducing measurement variability and enhancing internal validity. Children with mild ASD are also more likely to engage in structured school- or community-based exercise settings, increasing the translational relevance of the findings [17,21,22,23]. Furthermore, limited studies have implemented clearly structured, progressive resistance training protocols with defined frequency, intensity, and progression in children with ASD, limiting reproducibility and clinical translation. Therefore, the aim of the present study was to evaluate and compare the effects of a 12-week, age-appropriate resistance training program (versus an online-based stretching/yoga program) on muscular strength and functional performance in children aged 9–11 years presenting with mild ASD.

## 2. Materials and Methods

### 2.1. Participants and Study Design

A minimum sample size of 28 participants (i.e., 14 per group) was determined through a priori calculation using the following inputs: (i) a type-I error rate of 0.05, (ii) a type-II error rate of 0.20, (iii) the two groups (exercise vs. control), and (iv) a moderate effect size (partial eta-squared of 0.06). Sample size estimation was performed using G*Power software (version 3.1.9.7; Heinrich-Heine-Universität Düsseldorf, Germany). A selected-group repeated-measures study design was employed. Participants were allocated to the exercise or control group using a matched allocation procedure based on baseline strength values, rather than true randomization, to improve baseline equivalence in a small sample. Matching was performed by ranking participants according to baseline handgrip strength and allocating them alternately to each group to achieve comparable strength distributions. Where necessary, additional functional measures, such as vertical jump performance, were considered to maintain group equivalence. Baseline equivalence between groups was subsequently evaluated using statistical comparisons [28]. The selection of a moderate effect size was informed by previous pediatric exercise intervention studies reporting small-to-moderate strength adaptations following short-term resistance training programs in children [1,3].

Eligibility was determined based on specific inclusion and exclusion criteria. Participants were required to be between 9 and 11 years old, have a specialist-confirmed diagnosis of mild ASD (score ≤ 2 on the three-level severity ranking scale), and be able to walk and communicate verbally without assistance. To minimize miscommunication, only first-language English speakers were included. Participants provided informed parental consent and personal assent before enrolment. Those on chronic medication for ASD-related conditions, without other comorbidities, were eligible, as the study accounted for relevant exercise and testing considerations. Participants with known comorbidities such as cardiovascular, pulmonary, or metabolic diseases were excluded.

The exercise group followed a 12-week resistance training intervention program, while the control group participated in a 30 min online yoga-based stretching session three times per week over the same intervention period. All participants completed a series of tests at baseline, week 6, and week 12 to determine whether there were any improvements in the following areas: functional mobility, coordination, and muscular strength and power. These assessments included a handgrip strength test to evaluate muscular strength, a vertical jump test to assess explosive power, and a 10 m unassisted walk test to evaluate functional mobility. This study was conducted in accordance with the Declaration of Helsinki and approved by the Health Research Ethics Committee of the Faculty of Health Sciences, North-West University (ethics number: NWU-00455-20-A1), as well as the regional Department of Education.

### 2.2. Measuring Instruments and Procedures

A hand dynamometer (CAMRY, South El Monte, CA, USA) was used to measure grip strength, allowing for comparisons against normative data. The device is equipped with a high-precision gauge sensor and automatically displays the maximum recorded strength. The hand dynamometer has demonstrated high reliability, with a correlation coefficient of 0.94 for the right hand and 0.95 for the left [29]. It is widely used in research on musculoskeletal strength in children with ASD, offering a reliable and valid method for assessing muscle force and torque [30,31]. It was calibrated prior to testing according to the manufacturer’s specifications. Testing was performed with the dominant hand, forearm straight, and elbow close to the body. The handle was adjusted to rest on the first metacarpal and the middle of the other four digits. Participants squeezed the handle as hard as possible, flexing their elbows to 90 degrees before releasing. The measurement was repeated three times, with the best score recorded. A 30-s rest was provided between trials.

All outcome measures were coordinated by the same trained assessment team at baseline, 6 weeks, and post-intervention to minimize inter-rater variability. Assessors were not blinded to group allocation, which may have introduced measurement bias, particularly in performance-based outcomes. Prior to data collection, assessors underwent familiarization and protocol training to standardize testing procedures, verbal instructions, demonstrations, and motivational encouragement across all participants and time points. Standardized verbal cues were used during testing to minimize variability in participant motivation and examiner influence. Despite these precautions, the potential influence of assessor expectations cannot be excluded.

The vertical jump test was preferred over the broad jump due to concerns about balance and stability. It is the most commonly used lower limb test for evaluating power, with a reliability score of 0.93 [32]. Participants stood side-on to a wall, reaching up to mark standing reach height with chalk. Keeping both feet flat, they jumped using arms and legs to reach as high as possible, marking the highest point. The difference between the two marks was measured and recorded, with the best of three attempts noted. A 30-s rest was provided between trials.

The 10-m walk test was used to evaluate functional mobility and walking performance and is a highly reliable measure, with intra-rater reliability of 0.95 and inter-rater reliability of 0.997 [33,34]. Two cones were placed 10-m apart, and participants were timed while walking unassisted between them. The test was repeated three times, with the fastest time recorded to the nearest 0.01 s. A 30-s rest was provided between trials. The fastest time across three trials was recorded to represent optimal functional walking performance. Although this approach emphasizes speed, it is commonly used in pediatric and neurodevelopmental research to capture functional mobility capacity under standardized conditions. As the protocol was applied consistently across all participants and time points, relative changes over time were considered clinically relevant within the context of the standardized testing protocol.

### 2.3. Intervention and Control Group

Participants in the control group participated in a 30-min online yoga-based stretching session three times per week throughout the 12-week intervention period. The yoga-based stretching sessions consisted of low-intensity static and dynamic stretching exercises targeting major muscle groups, simple balance-oriented poses, breathing exercises, and simple mobility activities adapted for children with ASD. Sessions followed a standardized online format and were designed to promote flexibility, coordination, and routine participation without progressive overload.

This intervention was selected to control for supervision, attention, and routine engagement, while minimizing substantial progressive overload or strength-specific adaptations. It is acknowledged that such an intervention may influence balance and coordination outcomes. Participants in the exercise group followed a 12-week supervised resistance exercise training program designed with guidance from the American College of Sports Medicine (ACSM) guidelines for resistance training in children and adolescents, with modifications to accommodate varying functional abilities [35]. The program targeted the hamstrings, quadriceps, core, upper back, shoulders, and arm muscles, with exercises varying each session to maintain engagement. The resistance exercise training sessions were scheduled thrice a week over the 12-week intervention period, each lasting approximately 40–45 min, beginning with a 5-min warm-up, followed by the prescribed exercises, incorporating rest and water breaks, and concluding with a cool-down to reduce soreness and fatigue. Exercises were performed for 2–3 sets of 8–12 repetitions using body weight, resistance bands, and light external resistance. Intensity and complexity were progressed individually based on tolerance and movement competency. The resistance exercise training sessions were conducted in person by the primary researcher, a qualified biokineticist, and a trained research assistant with a background in exercise science. Biokinetics is a regulated health profession in South Africa that utilizes individualized exercise therapy to promote physical function, prevent disease, and support rehabilitation. Attendance was recorded, and a minimum adherence rate of 80% was required for inclusion in the analysis.

Testing sessions were conducted at baseline, week 6, and week 12 of the intervention period. These sessions involved six exercise stations (e.g., jump test, 10-m walk test, and handgrip test), each supervised by a trained research assistant to ensure reliability. Upon arrival, research participants were divided into groups of up to five and rotated through the stations, where scores were recorded on individual data sheets, which were stored securely. Testing sessions lasted 60–90 min, with all equipment sanitized before and after use. Data was recorded on anonymized datasheets, which linked test results to participant ID numbers to ensure accuracy and minimize human error during data entry.

### 2.4. Statistical Analysis

Data were analyzed using Jamovi statistical software (Version 2.5, The Jamovi Project). The Shapiro-Wilk test was employed to assess the normality of the data distribution. Given the small sample size (*n* = 28) and observed violations of normality for multiple variables, non-parametric statistical procedures were deemed appropriate to maintain statistical validity.

To assess within-group changes over time, the Friedman test was conducted. Where significant main effects were observed, post hoc Wilcoxon signed-rank tests were performed for pairwise comparisons, with rank-biserial correlation reported as the measure of effect size.

To compare between-group differences (control vs. exercise) at specific time points, the Mann-Whitney U test was employed. Effect sizes for these comparisons were reported using rank-biserial correlation to provide insight into the magnitude of between-group differences.

While the initial power calculation was based on a parametric repeated-measures ANOVA (as is common in pilot study designs), the shift to non-parametric analyses was justified to preserve statistical validity in light of the small sample size and non-normal data distribution. The authors acknowledge that non-parametric analyses generally have lower statistical power than parametric approaches, increasing the possibility of Type II error and potentially reducing the power to detect smaller effects. Therefore, effect sizes were interpreted alongside *p*-values to support clinical interpretation of the findings.

Given the exploratory nature and limited sample size, formal family-wise error corrections were not applied; however, effect sizes were reported to contextualize statistical significance.

## 3. Results

### 3.1. Descriptive Data

Table 1 summarizes the participant heights and body masses for the control and exercise groups, respectively, showing no significant differences at baseline between the groups. All references to time points have been standardized to “week 6” and “week 12” for consistency.

### 3.2. Handgrip Strength

A non-parametric Friedman test indicated no significant main effect for handgrip strength over time, χ^2^(5) = 6.86, *p* = 0.231, suggesting that changes across time points were not statistically significant when considering the overall model. Exploratory pairwise comparisons were nevertheless conducted to examine potential changes between specific time points in light of the small sample size and exploratory nature of the study.

**Figure 1:**
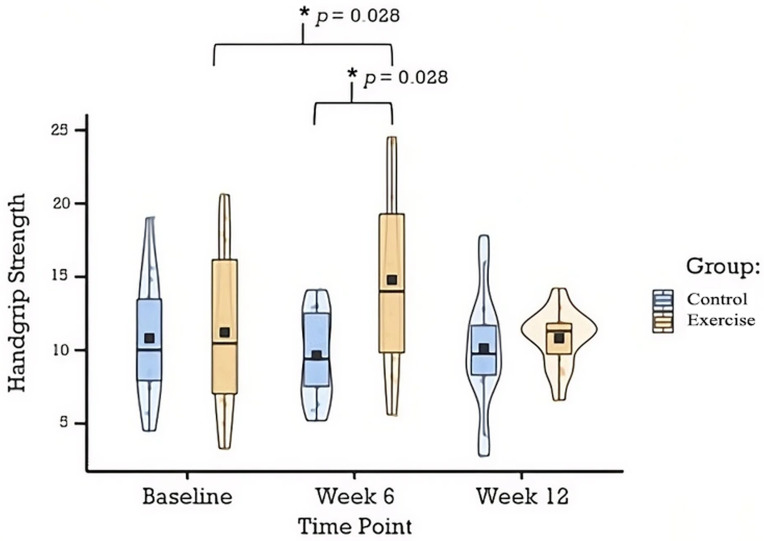
The changes in Handgrip Strength over the duration of the exercise intervention. * indicates a statistically significant within-group difference from baseline (*p* < 0.05). Between-group comparisons were performed at each time point using the Mann-Whitney U test.

Between-group comparisons conducted using the Mann-Whitney U test (Table 2) revealed no significant difference at baseline (Baseline: *U* = 96.0, *p* = 0.945, rank-biserial correlation = 0.020). An exploratory between-group difference was observed at week 6, favoring the exercise group (*U* = 48.5, *p* = 0.024, rank-biserial correlation = 0.505), indicating a moderate effect. By week 12, the between-group difference was no longer statistically significant (*U* = 83.0, *p* = 0.505, rank-biserial correlation = 0.153), indicating reduced between-group differences by week 12 (Figure 1).

**Table 2:** Mann–Whitney U test results for between-group differences in Handgrip Strength, Vertical Jump Height, and Walk Time.

Variable	Time Point	U Statistic	*p*-Value	Effect Size (Rank-Biserial)
Handgrip strength (kg)	Baseline	96.0	0.945	0.020
	Week 6	48.5	0.024 *	**0.505**
	Week 12	83.0	0.505	0.153
Vertical jump height (cm)	Baseline	80.5	0.433	0.179
	Week 6	56.0	0.056	**0.429**
	Week 12	84.5	0.549	0.138
Walk time (s)	Baseline	74.5	0.290	0.240
	Week 6	68.0	0.175	0.306
	Week 12	57.0	0.063	**0.418**

**Note:** U = Mann-Whitney U statistic. *p*-values are exact and two-tailed. Rank-biserial correlation (r) is reported as an effect size. Effect sizes were interpreted using Cohen’s conventions: small (r ≈ 0.1), moderate (r ≈ 0.3), and large (r ≥ 0.5). * *p* < 0.05. Bold values indicate moderate-to-large effect sizes (r ≥ 0.3). cm = centimeters; kg = kilograms; s = seconds.

### 3.3. Vertical Jump Height

A non-parametric Friedman test revealed a statistically significant main effect of time on vertical jump height, χ^2^(5) = 13.00, *p* = 0.024.

Within-group comparisons using Wilcoxon signed-rank tests (Table 3) showed a statistically significant improvement in the exercise group from baseline to week 6 (W = 0.0, *p* = 0.001, *r* = –1.000). No further significant changes were observed from week 6 to week 12 (W = 50.0, *p* = 0.900). The control group showed no significant changes across time points.

**Table 3:** Within-group changes in functional outcomes over time: Wilcoxon signed-rank test results.

Variable	Group	Comparison	W	*p*-Value	Rank-Biserial r
Handgrip strength (kg)	Exercise	W0 vs. W6	0.0	<0.001	−1.000
		W6 vs. W12	90.0	0.017	0.714
Vertical jump height (cm)	Exercise	W0 vs. W6	0.0	0.001	−1.000
Walk time (s)	Control	W0 vs. W6	94.0	0.007	0.790
		W0 vs. W12	90.0	0.017	0.714
	Exercise	W0 vs. W6	101.0	<0.001	0.924
		W6 vs. W12	103.0	<0.001	0.962

**Note:** Pairwise comparisons should be interpreted as exploratory due to the non-significant omnibus result for certain variables and the limited sample size. W = Wilcoxon signed-rank test statistic, based on the sum of signed ranks. *p*-values are exact and two-tailed. Only statistically significant comparisons (*p* < 0.05) are shown. Rank-biserial correlation (r) is reported as the effect size. Effect sizes were interpreted using Cohen’s conventions: small (r ≈ 0.1), moderate (r ≈ 0.3), and large (r ≥ 0.5). W0 = Week 0 (Baseline); W6 = Week 6; W12 = Week 12; kg = kilogram; cm = centimeter; s = seconds.

**Figure 2:**
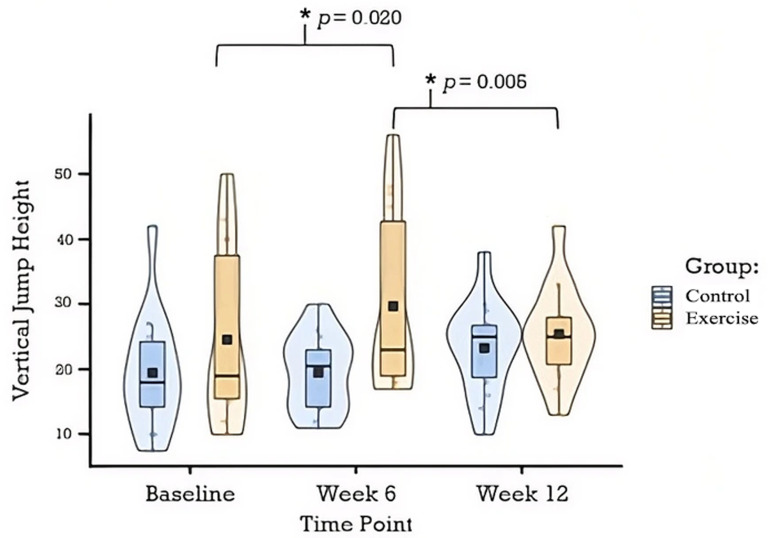
The changes in Vertical Jump Height over the duration of the exercise intervention. * indicates a statistically significant within-group difference from baseline (*p* < 0.024). Between-group comparisons were performed at each time point using the Mann–Whitney U test.

Between-group comparisons using Mann-Whitney U tests (Table 2) showed no significant difference between groups at baseline (U = 80.5, *p* = 0.433, *r* = 0.179). At week 6, the between-group difference did not reach statistical significance (U = 56.0, *p* = 0.056), with a moderate effect size (*r* = 0.429). No significant between-group differences were observed at week 12 (U = 84.5, *p* = 0.549, *r* = 0.138) (Figure 2).

### 3.4. Walk Time

A non-parametric Friedman test showed a statistically significant main effect of time on walk time, χ^2^(5) = 26.80, *p* < 0.001.

Within-group comparisons using Wilcoxon signed-rank tests (Table 3) revealed significant improvements in both groups.

In the exercise group, walk time decreased significantly from baseline to week 6 (W = 101.0, *p* < 0.001, r = 0.924) and from baseline to week 12 (W = 103.0, *p* < 0.001, r = 0.962), both with very large effect sizes. No significant change was observed between week 6 and week 12 (W = 78.0, *p* = 0.119).

In the control group, walk time also improved significantly from baseline to week 6 (W = 94.0, *p* = 0.007, r = 0.790) and from baseline to week 12 (W = 90.0, *p* = 0.017, r = 0.714). No significant change was observed between week 6 and week 12 (W = 76.0, *p* = 0.149).

**Figure 3:**
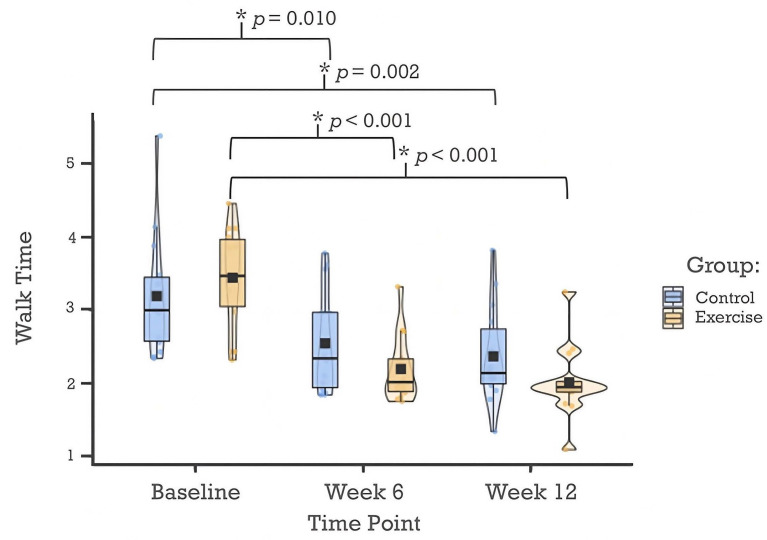
The changes in the 10 m Walk Time over the duration of the exercise intervention. * indicates a statistically significant within-group difference from baseline (*p* < 0.01). Between-group comparisons were performed at each time point using the Mann–Whitney U test.

Between-group comparisons using the Mann-Whitney U test (Table 2) showed no significant differences at baseline (U = 74.5, *p* = 0.290, *r* = 0.240) or week 6 (U = 68.0, *p* = 0.175, *r* = 0.306). At week 12, the between-group comparison did not reach statistical significance (U = 57.0, *p* = 0.063, *r* = 0.418), although a moderate effect favoring the exercise group was observed (Figure 3).

## 4. Discussion

The findings of this study demonstrate that a structured resistance training program can elicit meaningful improvements in muscular strength and functional performance in children with mild ASD. However, the pattern of adaptation observed across outcomes suggests that these responses are not uniform and may be influenced by individual variability, training exposure, and the nature of the outcome measures.

One of the primary findings is the significant improvement in handgrip strength among participants in the exercise group. Handgrip strength is a widely accepted surrogate marker of general muscular fitness and has been associated with better functional capacity and overall health status in both children and adults [36]. In this study, exploratory pairwise comparisons suggested improvements in handgrip strength by week 6 within the exercise group, although the overall omnibus effect across time points was not statistically significant. These findings align with previous research showing that grip strength is particularly responsive to resistance-based stimuli, likely due to its reliance on motor unit recruitment and neuromuscular coordination. While some studies in typically developing children and those with developmental delays report early-phase gains that plateau over time, some improvements in the current study appeared to persist at week 12. However, the reduction in between-group differences over time suggests that training adaptations may not have been consistently maintained across participants [36].

Increases in vertical jump height are indicative of improved lower-body explosive strength, neuromuscular coordination, and postural control, outcomes particularly relevant for children with ASD, who often present with hypotonia, motor planning difficulties, and gross motor delays [37]. In the current study, participants in the exercise group showed significant gains at week 6, aligning with the pediatric literature that reports early-phase improvements following resistance-based interventions [38]. This rapid response likely reflects enhanced motor unit recruitment and neuromuscular adaptation. However, these gains were not sustained at week 12, suggesting a possible plateau in adaptation responses, potentially due to inconsistent overload, motivation variability, or contextual factors affecting session fidelity. Although the between-group comparison at week 6 did not reach statistical significance, a moderate effect size favoring the exercise group was observed [37,38]. Importantly, variability in response to the intervention was observed, with some participants demonstrating greater improvements than others. This may be explained by differences in underlying neuromotor function, cognitive processing, and visuomotor integration, which are known to influence motor learning and adaptation in children with ASD. Previous research has highlighted the role of visuomotor processes in determining motor performance outcomes, suggesting that improvements may depend not only on physical training but also on individual neurocognitive characteristics.

Children with ASD frequently experience impairment in motor coordination, muscle tone, postural control, and strength [39]. These motor delays can discourage participation in physical activity, increase the risk of sedentary behavior, and limit opportunities for social interaction and physical development. Improvements in muscular strength may, therefore, have effects that extend beyond physical fitness: increased strength could translate to better performance in daily functional tasks, enhanced participation in physical education, and increased self-confidence in social environments.

Improvements in walking time were observed in both the exercise and control groups, particularly from baseline to week 6 and week 12. This suggests that repeated exposure to structured movement, whether through resistance-based training or stretching-focused control activities, may benefit functional mobility. These findings reinforce that the control intervention was not inert and likely offered a meaningful stimulus, which is in line with the previous literature in the field [20,24,31,38]. Consequently, the absence of statistically significant between-group differences should be interpreted cautiously, particularly considering the moderate effect size observed at week 12. An important consideration is that the control group participated in a structured yoga-based stretching program, making it an active control rather than a passive comparator. This likely contributed to improvements in walking performance observed in both groups and complicates the interpretation of between-group differences. It is therefore possible that both interventions were beneficial but influenced different aspects of physical function.

The structure of the experimental resistance exercise program employed in the current study, which was conducted in a supervised, progressive, and age-specific manner, appears to be effective and safe. No injuries or side effects were reported throughout the intervention, supporting the contention of the ACSM that resistance training can be safely implemented in pediatric populations, provided it is appropriately designed and supervised [35]. The control group, which did not participate in structured resistance exercise but instead completed a 30-min online yoga-based stretching program three times per week over the 12-week intervention period, showed no statistically significant changes in muscular strength outcomes. A few participants in the exercise group experienced slight decreases in vertical jump and grip strength between weeks 6 and 12, but these changes were not statistically significant. These findings may reflect a plateau in neuromuscular adaptation resulting from insufficient progressive overload during the latter stages of the intervention. External factors such as disruptions in routine and variable participant engagement may also have contributed to reduced responsiveness between weeks 6 and 12. The findings support the potential value of planned, targeted resistance-based physical activity in children with ASD, particularly for improving muscular performance outcomes. The results of the current study are in line with previous intervention research demonstrating the general benefits of exercise in children with ASD. Pitetti et al. [40] observed improvements in body composition and walking endurance following treadmill-based interventions. At the same time, Ketcheson et al. [39] demonstrated improvements in motor skills in young children with ASD following a motor skill intervention program. Similarly, Todd and Reid [41] found that increasing physical activity levels in individuals with autism not only improved physical outcomes but also helped to regulate behavioral and emotional states. Explanations for observed changes are therefore presented as plausible interpretations rather than confirmed mechanisms, as no direct physiological or neurological measures were included.

Our results have implications for educators, clinicians, therapists, and parents. The incorporation of resistance-based training into physical education, therapy, or after-school programs is a feasible, cost-effective way of enhancing physical fitness and developmental outcomes in children with ASD. In addition, improvements in physical capacities can lead to increased engagement in sports and recreation activities, which, in turn, can reduce social isolation, anxiety, and behavioral issues characteristic of this population.

While this study focused on physical outcomes, ASD is a multidimensional condition affecting cognitive, behavioral, and emotional domains. Resistance training may influence these areas indirectly through mechanisms such as improved self-regulation, sensory integration, and confidence. The absence of these measures in the current study limits the ability to interpret the broader impact of the intervention.

Although the results are promising, they should be interpreted in light of several limitations, particularly the relatively small sample size, which may limit generalizability and increase sensitivity to individual variability in training response. Small sample sizes are more susceptible to individual differences, which may exaggerate or obscure true intervention effects. As a result, the observed outcomes should be considered preliminary and require confirmation in larger, more heterogeneous samples.

Participants were limited to children with mild ASD to ensure task compliance and safety; however, this restricts the generalizability of the findings. Children with more severe ASD may respond differently due to greater cognitive, behavioral, and motor impairments, and these findings should therefore not be extrapolated beyond similar populations. In addition, assessors were not blinded to group allocation, which may have introduced measurement bias in performance-based outcomes despite the use of standardized instructions and testing procedures.

The study also relied on a limited battery of physical outcome measures, including handgrip strength, a 10-m walk test, and vertical jump height. While these measures are clinically relevant, they do not capture the full range of motor, behavioral, cognitive, and functional characteristics associated with ASD. Future studies should incorporate broader multidimensional outcome measures to better understand the wider effects of structured exercise interventions in this population.

## 5. Conclusions

This study provides preliminary evidence that a structured, age-appropriate resistance training program may support improvements in muscular strength and functional performance in preadolescent children with mild autism spectrum disorder. Improvements were most apparent during the early phases of the intervention, particularly for handgrip strength and vertical jump performance, although variability in responsiveness was observed across participants and outcomes.

Improvements in walk time were observed in both the exercise and control groups, suggesting that structured movement-based interventions more broadly may benefit functional mobility in children with ASD. Nevertheless, the moderate effect sizes observed for several muscular performance outcomes support the potential value of resistance-based exercise as part of broader physical activity programming in this population.

Given the exploratory nature and limited sample size of the current study, further research with larger and more diverse samples, longer intervention periods, and multidimensional outcome measures is required to better understand the long-term effects and clinical relevance of resistance training in children with ASD.

## Figures and Tables

**Table 1:** Descriptive data of the research participants.

Variable	Control Group (Mean [SD])	Exercise Group (Mean [SD])	*p*-Value
Height (cm)	136 [18.0]	142 [18.6]	0.366
Body Mass (kg)	47.6 [6.49]	49.9 [11.6]	0.518

**Note:** cm = centimeter, kg = kilogram, *p*-value = independent *t*-test, SD = standard deviation.

## Data Availability

The data supporting the findings of this study are available from the corresponding author upon reasonable request.

## Abbreviations

The following abbreviations are used in this manuscript:

ACSM	American College of Sports Medicine
ASD	Autism Spectrum Disorder
cm	Centimeter
CNS	Central Nervous System
EMG	Electromyography
HREC	Health Research Ethics Committee
kg	Kilogram
m	Meter
min	Minute
NWU	North-West University
s	Second(s)
SD	Standard Deviation
QoL	Quality of life
